# Novel Biallelic *NSUN3* Variants Cause Early-Onset Mitochondrial Encephalomyopathy and Seizures

**DOI:** 10.1007/s12031-020-01595-8

**Published:** 2020-06-02

**Authors:** Arumugam Paramasivam, Angamuthu K. Meena, Challa Venkatapathi, Robert D.S. Pitceathly, Kumarasamy Thangaraj

**Affiliations:** 1 CSIR-Centre for Cellular and Molecular Biology, Hyderabad, India; 2 BRULAC-DRC, Saveetha Dental College and Hospital, Saveetha Institute of Medical and Technical Sciences, Saveetha University, Chennai, India; 3 Department of Neurology, Nizam’s Institute of Medical Sciences, Hyderabad, India; 4 Department of Neuromuscular Diseases, UCL Queen Square Institute of Neurology and The National Hospital for Neurology and Neurosurgery, London, United Kingdom

**Keywords:** Epitranscriptomics, Mitochondrial disorders, mtDNA, *NSUN3*, Encephalomyopathy, Seizures

## Abstract

Epitranscriptomic systems enable post-transcriptional modifications of cellular RNA that are essential for regulating gene expression. Of the ~ 170 known RNA chemical modifications, methylation is among the most common. Loss of function mutations in *NSUN3*, encoding the 5-methylcytosine (m5C) methyltransferase NSun3, have been linked to multisystem mitochondrial disease associated with combined oxidative phosphorylation deficiency. Here, we report a patient with early-onset mitochondrial encephalomyopathy and seizures in whom the novel biallelic *NSUN3* missense variants c.421G>C (p.A141P) and c.454T>A (p.C152S) were detected. Segregation studies and in silico functional analysis confirmed the likely pathogenic effects of both variants. These findings expand the molecular and phenotypic spectrum of *NSUN3*-related mitochondrial disease.

## Introduction

Epitranscriptomic systems are necessary for the post-transcriptional modification of cellular RNA, RNA splicing and protein translation (Hsu et al. 2017; Rozov et al. 2016). Of the ~ 170 RNA chemical modifications reported, methylation is among the most common (Machnicka et al. 2013), with more than 90 reactions involving tRNA molecules (Boccaletto et al. 2018; Hussain et al. 2016). Numerous RNA modification enzymes and catalytic RNA-protein complexes are necessary for the post-transcriptional modification events (Rozov et al. 2016), although many remain poorly characterised.

Human diseases linked to defects in these pathways emphasise the important role of epitranscriptomics in gene expression (Hsu et al. 2017). One recent example involves the 5-methylcytosine (m5C) methyltransferase NSun3, encoded by NOP2/Sun RNA methyltransferase 3 (*NSUN3*). Loss of function mutations in *NSUN3* cause multisystem mitochondrial disease associated with a combined oxidative phosphorylation (OXPHOS) deficiency (Haag et al. 2016), highlighting the importance of NSun3 in mitochondrial translation.

Here, we report novel biallelic *NSUN3* missense variants in a South Asian patient with early-onset mitochondrial encephalomyopathy and seizures.

## Patients and Methods

### Patient Cohort

A cohort comprising 167 South Asian patients with suspected primary mitochondrial disease (PMD), based on clinical, pathological and biochemical findings, was included. Forty-eight patients had early-onset mitochondrial encephalomyopathy. Informed written consent for diagnostic and research studies was obtained from all subjects. The study was approved by the Institutional Ethical Committee (IEC) of CSIR-Centre for Cellular and Molecular Biology, Hyderabad, India, and Nizam’s Institute of Medical Sciences (NIMS), Hyderabad, India.

### Mitochondrial DNA Sequencing

DNA was extracted from blood leucocytes using a standard phenol-chloroform method (Thangaraj et al. 2002), with minor modifications. Complete mtDNA was amplified using 24 sets of primers to generate overlapping amplicons, purified and directionally sequenced using BigDye terminator cycle sequencing kit and ABI3730 XL Genetic Analyzer (Rieder et al. 1998).

### Analysis of Mitochondrial-Related Nuclear Genes

All mitochondrial nuclear genes (*POLG, POLG2, TK2, SLC25A4, DGUOK, MPV17, RRM2B, MFN2, SPG7, AFG3L2, RNASEH1* and *NSUN3*) were analysed using custom-designed primers (http://frodo.wi.mit.edu) and bidirectionally Sanger sequenced using BigDye terminator cycle sequencing kit and 3730XL Genetic Analyzer. DNA variations were identified after assembling each patient’s sequence with the reference sequence using Auto-assembler software (Applied Bio-systems).

### In Silico Functional Analysis

To evaluate the potential functional impact of the identified missense variants, we utilised a variety of pathogenicity prediction programs, including SIFT (http://sift.jcvi.org/), PolyPhen-2 (http://genetics.bwh.harvard.edu/pph2//) and MutationTaster (http://www.mutationtaster.org/).

## Results

**Fig. 1:**
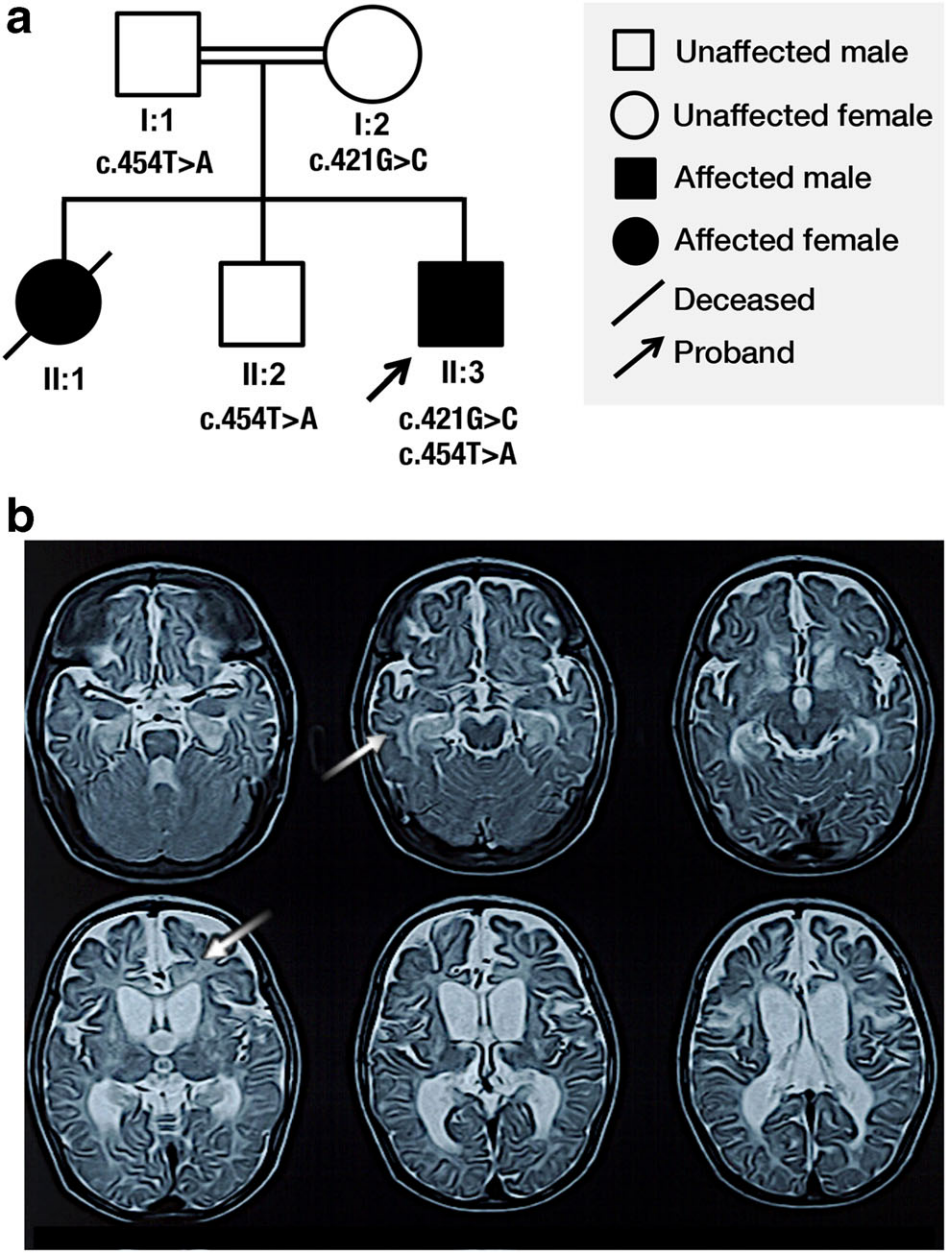
Pedigree demonstrating segregation of the novel *NSUN3* variants (**a**). Proband’s unaffected father (I:1). Proband’s unaffected mother (I:2). Proband’s elder sister (II:1). Proband’s elder brother (II:2). Proband (II:3). T2-weighted axial images demonstrate white matter hyperintensities in both cerebral hemispheres, predominantly affecting the frontal and temporal lobes, arrows (**b**)

The novel compound heterozygous *NSUN3* missense variants c.421G>C (p.A141P) and c.454T>A (p.C152S) were detected in one patient with early-onset mitochondrial encephalomyopathy and seizures (Fig. 1a, II:3). The proband, an 8-month-old boy born to consanguineous parents following a normal pregnancy, presented at 4 months of age with lactic acidosis, global developmental delay, hypotonia, muscle weakness and seizures. There was a family history of lactic acidosis in the proband’s elder sister (Fig. 1a, II:1). Brain magnetic resonance imaging (MRI) revealed cerebral atrophy and white matter hyperintensities involving both cerebral hemispheres, particularly the frontal and temporal lobes (Fig. 1b). Segregation studies confirmed that the proband’s unaffected father (Fig. 1a, I:1) and elder brother (Fig. 1a, II:2) were carriers of the c.454T>A (p.C152S) variant, while the proband’s unaffected mother (Fig. 1a, I:2) was a carrier for the c.421G>C (p.A141P) variant.

**Fig. 2:**
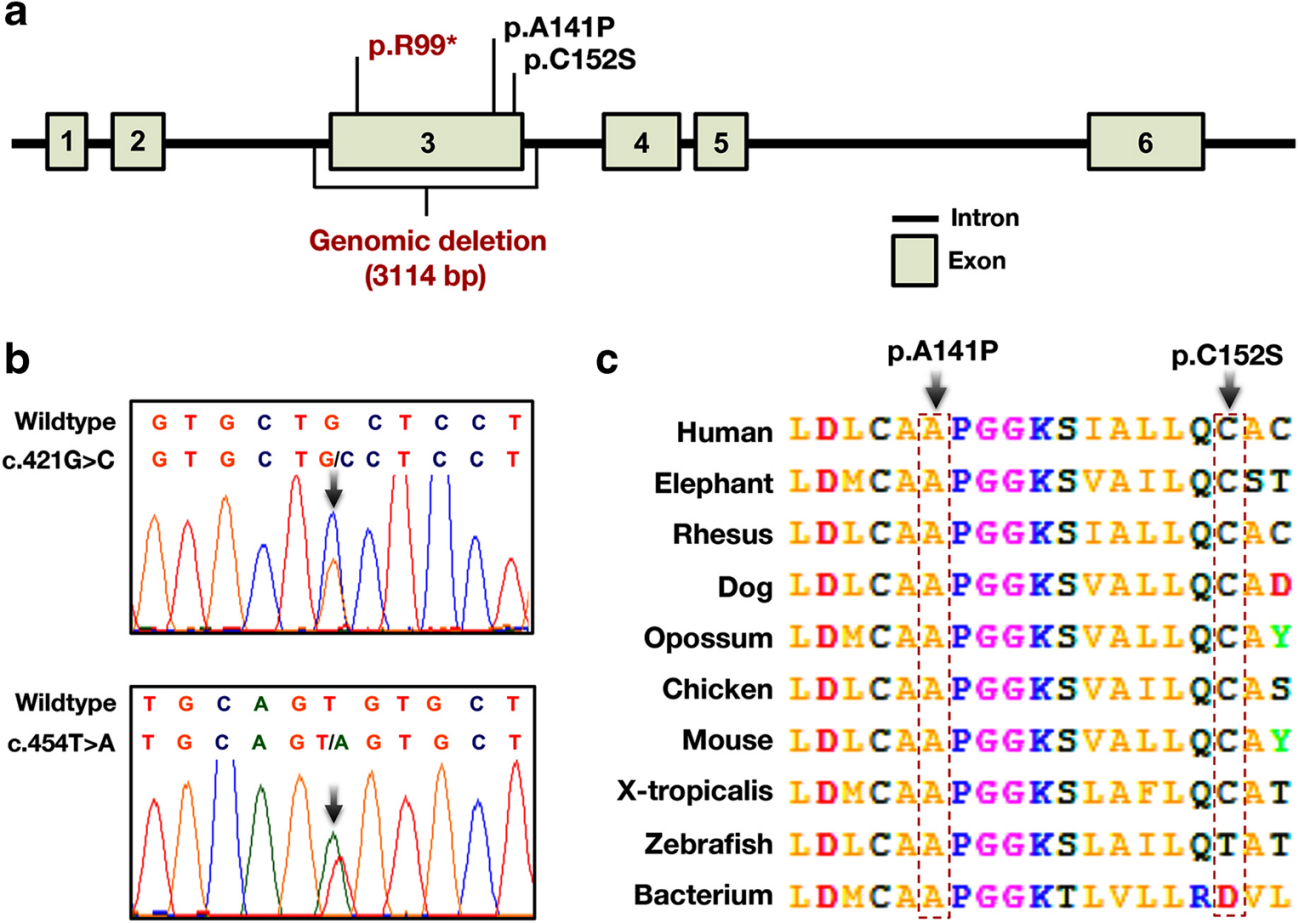
Schematic diagram of the *NSUN3* gene with previously reported variants (red letters) and the novel variants identified in this study (black letters) annotated (**a**). Sequence analysis of *NSUN3* in the proband confirming the c.421G>C (p.A141P) and c.454T>A (p.C152S) *NSUN3* variants, arrows (**b**). Evolutionary conservation data for NSun3 amino acid sequence across species at positions 141 and 152, arrows (**c**)

Both variants reside in exon 3 of the *NSUN3* gene (Fig. 2 a and b), within a highly conserved region of the protein (Fig. 2c), and are absent in the 1000 Genomes Project (http://www.1000genomes.org/about), Exome Aggregation Consortium (ExAC, http://exac.broadinstitute.org/), Genome Aggregation Database (gnomAD, http://gnomad-old.broadinstitute.org/) and 485 ethnically matched healthy control subjects. In silico functional analysis confirmed that the variants have a high probability of being pathogenic (Table 1). Unfortunately, muscle tissue and cultured fibroblasts were unavailable for biochemical or functional studies.

**Table 1:** Summary of the in silico functional analysis, open access genetic databases and in-house ethnically matched healthy control subjects used to evaluate the reported *NSUN3* variants

Mutation	Amino acid change	SIFT	Polyphen-2	Mutation Taster	ExAC	EVS	1000 Genomes	HGVD	gnomAD	*Controls *n* = 485
c.421G>C	p.A141P	Deleterious (score 0.00)	Probably Damaging	Disease causing	No	No	No	No	No	No
c.454T>A	p.C152S	Tolerated (score 0.50)	Possibly Damaging	Disease causing	No	No	No	No	No	No

*ExAC* Exome Aggregation Consortium, *EVS* Exome Variant Server, *HGVD* Human Genetic Variation Database

*Ethnically matched healthy control subjects

## Discussion

We report a South Asian patient with early-onset mitochondrial encephalomyopathy and seizures harbouring novel, compound heterozygous *NSUN3* missense variants c.421G>C (p.A141P) and c.454T>A (p.C152S). *NSUN3* comprises six exons that encode a 340 amino acid protein. The c.421G>C and c.454T>A missense variants reside within exon 3 and result in substitutions of an alanine to proline (p.A141P) and a cysteine to serine (p.C152S), respectively. The two previously reported pathogenic *NSUN3* variants (c.123-615_466 + 2155del and c.295C>T) also occur in exon 3 (Van Haute et al. 2016). Thus, this region of the gene potentially represents a hotspot for *NSUN3*-related mitochondrial disease.

NSun3 is a novel human m5C RNA methyltransferase that specifically methylates mitochondrial tRNA^Met^. *NSUN3*-knockout cells demonstrate reduced mitochondrial protein synthesis and oxygen consumption resulting in decreased mitochondrial activity (Trixl et al. 2018). Disease-associated mt-tRNA^Met^ point mutations that impair Nsun3-mediated methylation have pathological consequences (Nakano et al. 2016), and loss of function mutations in *NSUN3* are reported to cause severe multisystem mitochondrial disease associated with combined OXPHOS deficiency (Van Haute et al. 2016). In the current study, we expand the molecular and clinical spectrum of *NSUN3*-related mitochondrial disease to include two novel *NSUN3* missense variants and seizures.

The following lines of evidence support the pathogenic effects of the c.421G>C (p.A141P) and c.454T>A (p.C152S) *NSUN3* variants: (1) conservation data suggests p.A141 and p.C152 are highly conserved amino acid residues of the Nsun3 protein; (2) both variants are absent from the 1000 Genomes Project, ExAC, gnomAD and 485 ethnically matched healthy control subjects; (3) segregation studies confirm the variants are trans-acting in the proband, while both parents and unaffected sibling are heterozygous carriers; (4) in silico functional analyses predict the variants to be pathogenic with high probability scores; and (5) no known pathogenic variants were detected in either mtDNA or disease-associated mitochondrial nuclear maintenance genes.

In conclusion, we report novel biallelic *NSUN3* missense variants causing early-onset mitochondrial encephalomyopathy and seizures, thereby expanding the molecular and phenotypic spectrum of *NSUN3*-related mitochondrial disease.
