# Correction to: Novel Biallelic *NSUN3* Variants Cause Early-Onset Mitochondrial Encephalomyopathy and Seizures

**DOI:** 10.1007/s12031-020-01652-2

**Published:** 2020-07-16

**Authors:** Arumugam Paramasivam, Angamuthu K. Meena, Challa Venkatapathi, Robert D. S. Pitceathly, Kumarasamy Thangaraj

**Affiliations:** 1 CSIR-Centre for Cellular and Molecular Biology, Hyderabad, India; 2 BRULAC-DRC, Saveetha Dental College and Hospital, Saveetha Institute of Medical and Technical Sciences, Saveetha University, Chennai, India; 3 Department of Neurology, Nizam’s Institute of Medical Sciences, Hyderabad, India; 4 Department of Neuromuscular Diseases, UCL Queen Square Institute of Neurology and The National Hospital for Neurology and Neurosurgery, London, UK

**Correction to: Journal of Molecular Neuroscience**

10.1007/s12031-020-01595-8

The article “Novel Biallelic *NSUN3* Variants Cause Early-Onset Mitochondrial Encephalomyopathy and Seizures”, written by Arumugam Paramasivam, Angamuthu K. Meena, Challa Venkatapathi, Robert D.S. Pitceathly and Kumarasamy Thangaraj, was originally published electronically on the publisher’s internet portal on 02 June 2020 without open access.

With the author(s)’ decision to opt for Open Choice the copyright of the article changed on July 2020 to © The Author(s) 2020 and the article is forthwith distributed under a Creative Commons Attribution 4.0 International License (https://creativecommons.org/licenses/by/4.0/), which permits use, sharing, adaptation, distribution and reproduction in any medium or format, as long as you give appropriate credit to the original author(s) and the source, provide a link to the Creative Commons license, and indicate if changes were made.

The original article has been corrected.

